# Analysis of Purines and Pyrimidines distribution over miRNAs of Human, Gorilla, Chimpanzee, Mouse and Rat

**DOI:** 10.1038/s41598-018-28289-x

**Published:** 2018-07-02

**Authors:** Jayanta Kumar Das, Pabitra Pal Choudhury, Adwitiya Chaudhuri, Sk. Sarif Hassan, Pallab Basu

**Affiliations:** 1 Applied Statistics Unit, Indian Statistical Institute, 203 B.T. Road, Kolkata, 700108 West Bengal India; 2 Department of Zoology, Pingla Thana Mahavidyalaya, Paschim Medinipur, 722140 West Bengal India; 3 Department of Mathematics, Pingla Thana Mahavidyalaya, Paschim Medinipur, 722140 West Bengal India; 4 International Centre for Theoretical Sciences, TIFR, Bangalore, 560089 Karnataka India

## Abstract

Meaningful words in English need vowels to break up the sounds that consonants make. The Nature has encoded her messages in RNA molecules using only four alphabets A, U, C and G in which the nine member double-ring bases (adenine (A) and Guanine (G)) are purines, while the six member single-ring bases (cytosine (C) and uracil (U)) are pyrimidines. Four bases A, U, C and G of RNA sequences are divided into three kinds of classifications according to their chemical properties. One of the three classifications, the purine-pyrimidine class is important. In understanding the distribution (organization) of purines and pyrimidines over some of the non-coding RNAs, all miRNAs of the three species (human, gorilla and chimpanzee) of Hominidae family and two species (mouse and rat) from of Muridae family are considered. The distribution of purines and pyrimidines over miRNA shows deviation from randomness. Based on the quantitative metrics (fractal dimension, Hurst exponent, Hamming distance, distance pattern of purine-pyrimidine, density distribution of purine-pyrimidine and Shannon entropy) five different clusters have been made for the five species. We have observed some couple of results including the closeness of different clusters among the five species.

## Introduction

The deoxyribonucleic acid (DNA) and the ribonucleic acid (RNA) are made of nucleotides (A/T or U/C/G), the monomer-units of nucleic acids. Nucleotides are grouped into three different classes based on their chemical properties, i.e., purine group R = {A, G} and pyrimidine group Y = {C, T/U}; amino group M = {A, C} and keto group K = {G, T/U}; strong H-bond group S = {C, G} and weak H-bond group W = {A, T/U}^1^. There are two kinds of nitrogen-containing bases - Purines and Pyrimidines, first isolated from hydrolysates of nucleic acids, were identified using classical methods of organic chemistry. An important contribution was made by Emil Fischer who must be credited with the earliest synthesis of purines (1897)^2^. Purines consist of a nine member double-ring (containing carbon and nitrogen) fused together, where as pyrimidines have a six member single-ring comprising of carbon and nitrogen^3,4^. Among three biological properties namely purine/pyrimidine, strong/week hydrogen bond and amino/keto, our analysis demonstrates a strong evidence that the organization of purine-pyrimidine bases over miRNAs is crucial. Therefore, we intend to understand the organization of the two chemical bases purine and pyrimidine over some of the non-coding RNAs, *microRNAs* using different mathematical parameters.

MicroRNAs (abbreviated miRNAs) contain about 18–25 ribonucleotides that can play important gene regulatory roles by pairing to the messages of protein-coding genes, to specify messenger RNA (mRNA) cleavage or repression of productive translation^5–7^. miRNA genes are one of the more abundant classes of regulatory genes in animals, estimated to comprise between 0.5 and 1 percent of the predicted genes in worms, flies, and humans, raising the prospect that they could have many more regulatory functions than those uncovered to date^8–10^. The main function of miRNAs is to down-regulate gene expression^11^. One miRNA may target several mRNAs, and a particular mRNA might be regulated by multiple miRNAs^12–17^. It is important to identify the miRNA targets accurately. miRNAs control gene expression by targeting mRNAs and triggering either translation repression or RNA degradation^18–20^. Their aberrant expression may be involved in various human diseases, including cancer^21–27^. miRNA regulatory mechanisms are complex and there is still no high-throughput and low-cost miRNA target screening technique^28–32^. It is an well known fact that each miRNA is potentially able to regulate around 100 or more target mRNAs and 30% of all human genes are regulated by miRNAs^33^.

In this article an attempt has been made to decipher the patterns of purine and pyrimidine distributions over the miRNAs of the three species human, gorilla and chimpanzee from Homonidae family and two species mouse and rat from Muridae family. We desire to understand how the purine and pyrimidine bases are organized over the sequence and how much distantly the purine or pyrimidine bases can be placed over the sequence. Which one of these two types of chemical bases purine or pyrimidine dominates the other in terms of their frequency density over the sequence is one of our prime aims to comprehend. A simple binomial distribution (i.e. location independent occurrence of the bases) fails to describe the observed variation of purine and pyrimidine. This encourages us to look for further patterns. We investigate, the self-organization of the purine and pyrimidine bases for all the miRNAs of the five species human, gorilla, chimpanzee, mouse and rat through the fractal dimension of the indicator matrix. The auto correlation of purine-pyrimidine bases over the miRNAs through the parameter Hurst exponent is determined and found many of the miRNAs having identical auto correlations even if their purine-pyrimidine organization is different. All the miRNAs are compared about their nearness based on their purine-pyrimidine distribution, Hamming distance is employed among all the miRNAs in understanding the nearness of purine-pyrimidine organization. The purine-pyrimidine distance patterns including the frequency distribution have been found for all the miRNAs for all the five species. All possible distinct patterns of frequency distribution are determined for all the miRNAs of all the five species. Here we wish to bring attention to the reader that through our investigation, the one miRNA *hsa-miR-6124 MIMAT0024597* of human, made of only purine bases is identified. There is no miRNA (human, gorilla, chimpanzee, mouse and rat) which is absolutely made of pyrimidines. In order to understand the association among miRNAs and their target mRNAs, we take a set of mRNAs from human species. Based on the quantitative measures, we have examined the set of miRNAs which relates the associations with the target mRNAs.

## Materials and Methods

### Dataset Specification

From the MiRBase (a miRNA database: http://www.mirbase.org/ (Release 21))^34^, from the family Hominidae, total of 2588 mature miRNAs of human, 357 mature miRNAs of gorilla and 587 mature miRNAs of chimpanzee and from the family Muriade, total of 1915 mature miRNAs of mouse and 765 miRNAs of rat are taken. Each miRNA of human, gorilla, chimpanzee, mouse and rat are encoded as numbers starting from *h*1 to the total number of sequences *h*2588 for miRNAs of human and same has been made for miRNAs of gorilla *g*1 to *g*357, for miRNAs of chimpanzee *p*1 to *p*587, for miRNAs of mouse *m*1 to *m*1915 and for miRNAs of rat *r*1 to *r*765 (Supplementary Table S1). We then transform the miRNAs sequences (A, U, C, G) into binary sequences (1’s and 0’s) according to the following rules:

$$\begin{array}{c}A/G\to \mathrm{1;}\\ U/C\to \mathrm{0;}\end{array}$$

That means purine and pyrimidine nucleotide bases are encoded as 1 and 0 respectively into the transformed binary sequences of miRNAs. Therefore, presently we have five datasets of binary sequences from the five species human, gorilla, chimpanzee, mouse and rat. All the computational codes are written in *MATLAB R2016a* software. One can easily obtain the results of the discussed methods of this article for any datasets, the detailed procedures are discussed and also we have provided the source codes (MATLAB 2016 onwards) in Supplementary Table S2.

### Fractal Dimension of Indicator Matrices

Here we shall encode each binary sequences into its indicator matrices^35,36^. It is noted that there are several other techniques for finding fractal dimension and self-organization structure of DNA sequences^37,38^. Consider a set **S** = {0, 1} and an indicator function *f* : {0, 1} × {0, 1} ↔ {0, 1} is defined as for all (*x*, *y*) ∈ **S** × **S**,

1 $$f(x,\,y)=(\begin{array}{cc}\mathrm{1,} & {\rm{if}}x=y\\ \mathrm{0,} & {\rm{if}}x\ne y\end{array}$$

**Figure 1:**
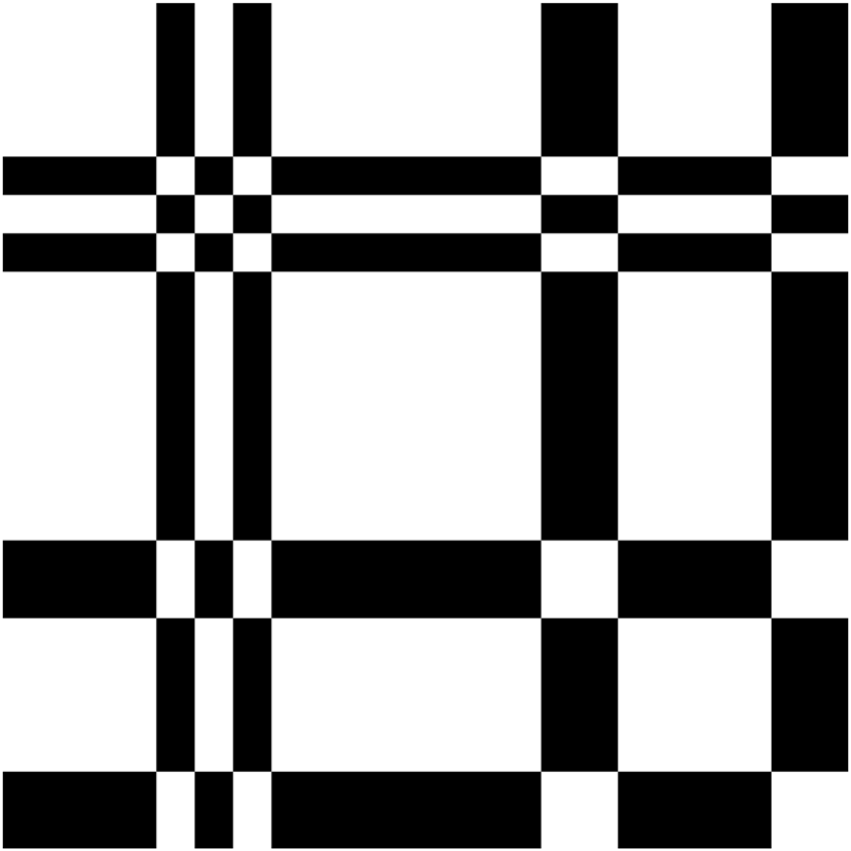
Indicator matrix for the binary sequence *Hsa*−*miR*−576−3*pMIMAT*0004796: 1111010111111100111100.

This indicator function can be used to obtain the binary image of the binary sequence as a two dimensional dot-plot. The binary image obtained by this indicator matrix can be used to visualize the distribution of ones and zeros within the same binary sequence and some kind of auto-correlation between the ones and zeros of the same sequence. It can be easily drawn by assigning a black dot to 1 and a white dot to 0. An example of indicator matrix is shown in Fig. 1 for the binary sequence *Hsa*−*miR*−576−3*pMIMAT*0004796: 1111010111111100111100.

From the indicator matrix, we can have an idea of the “fractal-like” distribution of ones and zeros (purines and pyrimidines). The fractal dimension for the graphical representation of the indicator matrix plots can be computed as the average of the number *p*(*n*) of 1 in the randomly taken *n* × *n* minors of the *N* × *N* indicator matrix. Using *p*(*n*), the fractal dimension (FD) is defined below.

2 $$FD=\frac{1}{N}\sum _{n=2}^{N}\frac{logp\,(n)}{log\,n}$$

The self-organization of the purine and pyrimidine bases for all the miRNAs can be obtained through the fractal dimension of the indicator matrix.

### Hurst Exponent of Binary Sequences

The Hurst Exponent (HE) deciphers the autocorrelation of a time series appeared in several areas of applied mathematics^39–41^. Hurst exponent ranges from 0 to 1. A value of *HE* in the interval [0, 0.5] indicates a time series with negatively autocorrelated and a value of *HE* in the interval [0.5, 1] indicates a time series with positively autocorrelated. A value of *HE* = 0.5 indicates a random series, there is no correlation of the variable with its past value. The larger the *HE* value is the stronger the correlation.

The Hurst exponent of a binary sequence {*x*_*n*_} is defined as

3 $${(\frac{n}{2})}^{HE}=\frac{R(n)}{S(n)}$$

where $S(n)=\sqrt{\frac{1}{n}{\sum }_{i}^{n}({x}_{i}-m)}$ and *R*(*n*) = *maxY* (*i*, *n*) − *minY* (*i*, *n*); 1 ≤ *i* ≤ *n* where $Y(i)={\sum }_{j=1}^{i}({x}_{j}-m)$ and $m=\sqrt{\frac{1}{n}{\sum }_{i=1}^{n}{x}_{i}}$

The auto correlation of purine-pyrimidine bases for all the miRNAs is obtained through the Hurst exponent.

### Hamming Distance of Binary Sequences

The Hamming Distance (*HD*) between two binary strings is the number of bits in which they differ^42–44^. Since length of the miRNAs might differ and hence a special care has been taken into consideration. Suppose there are two miRNAs *S*_*n*_ and *S*_*m*_ of length *n* and *m* respectively (*n* > *m*), then

4 $$HD({S}_{n},\,{S}_{m})=min(hd({S}_{n},{S}_{m}))$$

where *S*_*m*_ of length *m* window is sliding over *S*_*n*_ from the left alignment to the right alignment and each time hamming distance (hd) is calculated, and finally minimum *hd* value is taken as hamming distance *HD* of two binary sequences.

For example, take two binary sequences *S*_*n*_ = 010100 and *S*_*m*_ = 1101, now sliding of *S*_*m*_ over *S*_*n*_ of length 4, from left to right alignment of these two sequences, we find the hamming distances are hd(**0101**00,**1101**) = 1, hd(0**1010**0,**1101**) = 3, hd(01**0100**,**1101**) = 2, therefore we take *HD* = 1 (minimum) of these two binary sequences. Finding the minimum hamming distance of the two binary sequences says about the maximum similarity of two sequences over the distribution of purines and pyrimidines. The minimum value of *HD* = 0 when the pattern of length *min*(*n*, *m*) of two binary sequences of miRNAs are exactly identical i.e. similar distribution of purines and pyrimidines over the miRNAs of the two sequences and the maximum value of *HD* = *min*(*n*, *m*) when the pattern of length *min*(*n*, *m*) of two binary sequences of miRNAs are exactly opposite i.e. completely dissimilar distribution of purines and pyrimidines over miRNAs two sequences.

To get the nearness of the miRNAs based on their purine-pyrimidine distribution, minimum Hamming distance is deployed.

### Distance pattern of purine and pyrimidine over miRNAs

Here we are exploring the distance pattern of purines bases across the miRNAs of five species. How sparsely (closely) purine bases are placed over the miRNAs. So we find the distance (gap) between purine bases to the immediate next purine base over the miRNA sequences.

For example, take a transformed binary sequence *S*_*m*_ = 110100111000001, where 1 indicates the purines bases and 0 indicates pyrimidine bases in the sequence. From left to right the positions of 1’s and 0’s in serial is shown in Table 1. Now, from the distribution of 1’s, we find the purine distances at 1 (two consecutive 1’s at a distance of 1: 11), 2 (two consecutive 1’s at a distance of 2: 101), 3 (two consecutive 1’s at a distance of 3: 1001) and 6 (two consecutive 1’s at a distance of 6: 1000001). So, the distance pattern of purines (Purine-Distance pattern(Pu-Dp)) over the sequence is [1-2-3-6] in order.

**Table 1:** The position of each bit (1/0) of transformed binary sequence of *S*_*m*_ = 110100111000001 from left to right.

Positions	1	2	3	4	5	6	7	8	9	10	11	12	13	14	15
Sequence	1	1	0	1	0	0	1	1	1	0	0	0	0	0	1

Similar to the distance pattern of purine (Pyrimidine-Distance pattern(Py-Dp)), the distance pattern of pyrimidine bases (0’s) across the miRNAs also can be determined. The distance pattern of pyrimidines of the above sequence is [1-2-4] in order. Further the distance pattern of pyrimidines [1 **2 4**] of a miRNA opens up a fact that there is at least one 1(=**2**−1) and at least one 3(=**4**−1) length purine blocks present in the miRNA. In the similar way, a distance pattern of purine triggers the presence of pyrimidine blocks in miRNAs. If there is no distance pattern of purine (or pyrimidine) i.e. miRNA is made of only the pyrimidine (or purine) bases respectively, we denote the distance pattern of purine (or pyrimidine) as [−] and if the miRNA is having a single purine (or pyrimidine) base, we denote the distance pattern of purine (or pyrimidine) as [0].

### Shannon entropy of miRNAs

The Shannon entropy (SE) mesures information-entropy of a Bernoulli process with probability *p* of the two outcomes (0/1)^45–47^. It is defined as

5 $$SE=-\,\sum _{i=1}^{2}{p}_{i}lo{g}_{2}({p}_{i})$$

where ${p}_{1}=\frac{k}{{2}^{l}}$ and ${p}_{2}=\frac{l-k}{{2}^{l}}$; here *l* is length of the binary string and *k* is the number of 1’s in the binary string of length *l*.

The binary *Shannon entropy* is a measure of the uncertainty in a binary string. Whenever the probability *p* = 0, the event is certain never to occur, and so there is no uncertainty, leading to an entropy of 0. Similarly, if the probability *p* = 1, the result is certain, so the entropy must be 0. When *p* = 1/2, the uncertainty is at a maximum and consequently the SE is 1^45^.

## Results

### Deviation from randomness

A simple random binomial (p, q) model^48^, where each entries can either be purine (or pyrimidine) with probability *p* (or *q* = 1 − *p*) fails to address the distribution of purine or pyrimidine over miRNAs. We can calculate the mean ($\bar{x}$) of the distribution from the sample. If we divide the sample mean by the average sample size (*n*) we get a probability *p*. From this probability, we can calculate the expected variance *npq*. We can also calculate the variance for *m* number of samples *x*_1_, *x*_2_, …, *x*_*m*_ using $\frac{1}{m-1}{\sum }_{i=1}^{m}{({x}_{i}-\bar{x})}^{2}$. The standard deviation (std) is the square root of the variance.

For purine, in human *p* = 0.509, gorilla *p* = 0.514, chimpanzee *p* = 0.505, mouse *p* = 0.495 and rat *p* = 0.473 from mean. So, expected std = 2.323 (human), 2.322 (gorilla), 2.327 (chimpanzee), 2.340 (mouse) and 2.325 (rat).

The sample std = 3.42 (human), 2.64 (gorilla), 2.79 (chimpanzee), 3.48 (mouse) and 2.79 (rat).

So here we see that in all five species the expected variances through binomial distribution are significantly smaller than what we would have expected from the sample.

### Classification Based on FDs of Indicator Matrices

**Figure 2:**
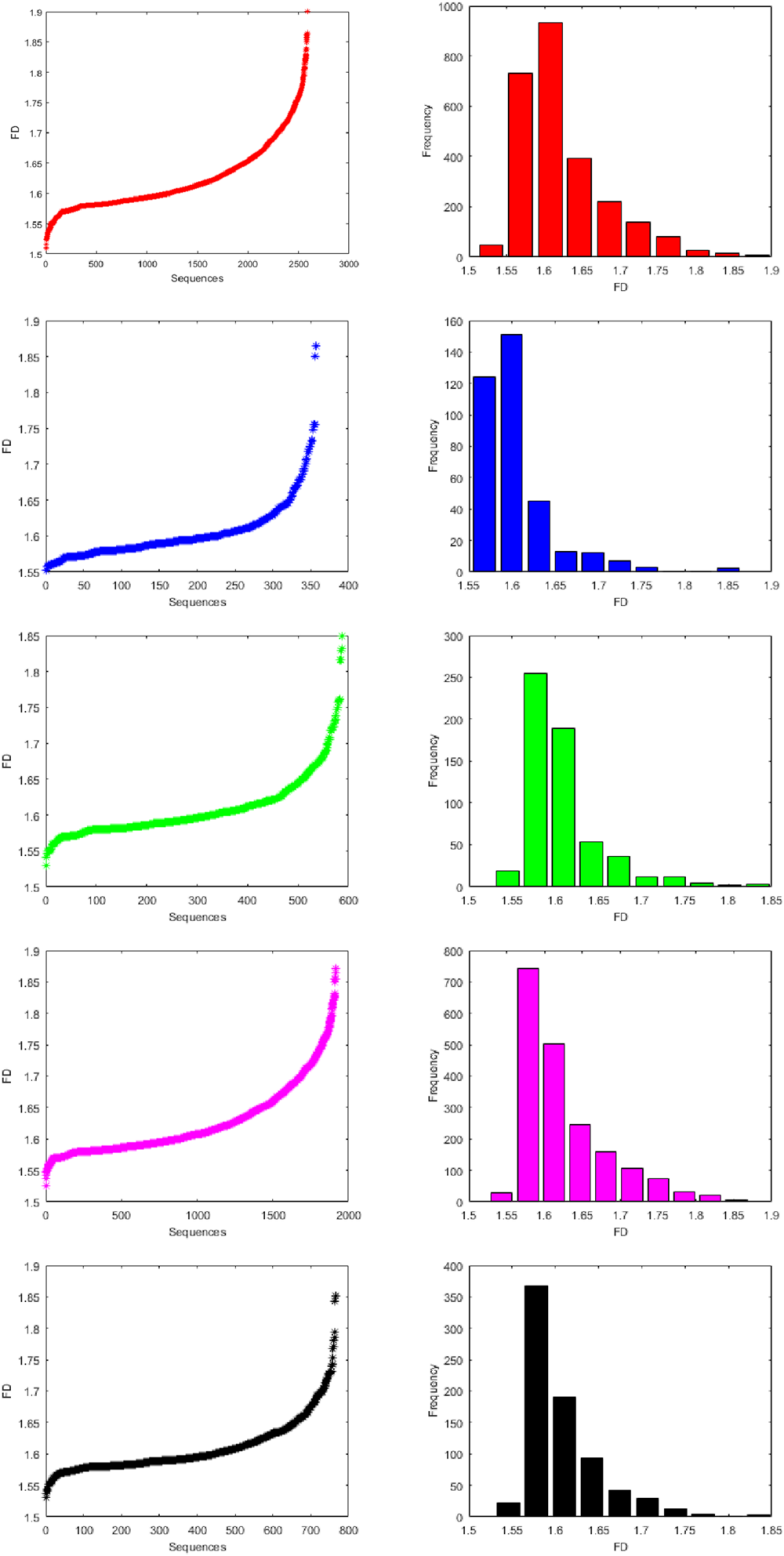
Histograms of fractal dimensions of miRNAs of Human, Gorilla, Chimpanzee, Mouse and Rat from top to bottom respectively.

**Figure 3:**
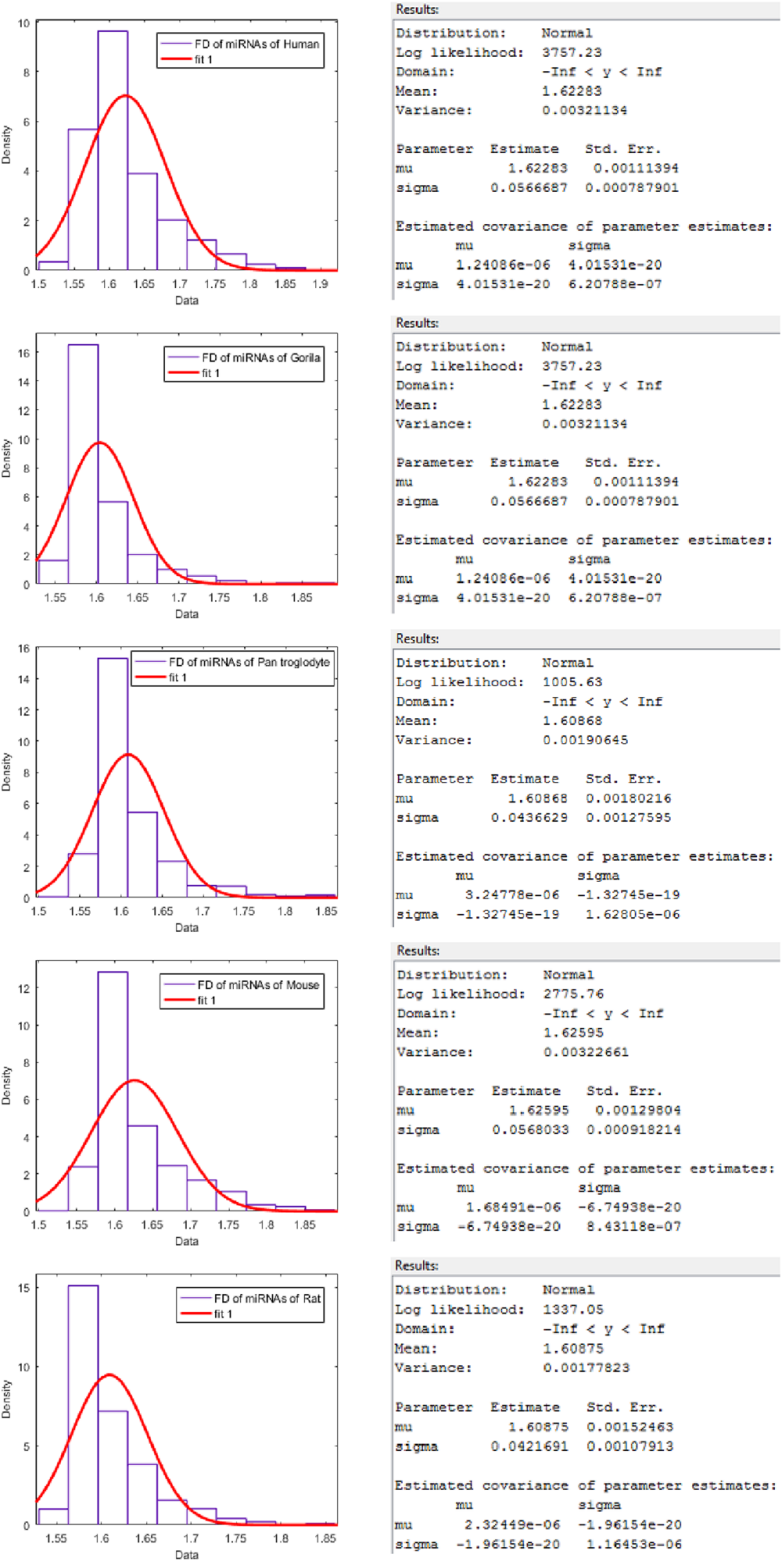
Normal distribution fitting over FDs of miRNAs of Human, Gorilla, Chimpanzee, Mouse and Rat from top to bottom respectively.

For each binary sequence of miRNA of human, gorilla, chimpanzee, mouse and rat, the fractal dimension (using Equation (2)) is calculated. Based on the fractal dimension, we have made classifications (clusters) for all the the datasets of the five species. There are 10 clusters of miRNAs of each species as shown in Table 2. The fractal dimensions including the histograms of all the miRNAs of human, gorilla, chimpanzee, mouse and rat are plotted in the Fig. 2. Also a normal distribution fitting is also made as shown in Fig. 3.

**Table 2:** Clusters based on Fractal dimension of miRNAs of Human, Gorilla and Chimpanzee, Mouse and Rat.

Cluster	Human		Gorilla		Chimpanzee		Mouse		Rat	
	Frequency	Center	Frequency	Center	Frequency	Center	Frequency	Center	Frequency	Center
1	48	1.529	124	1.568	19	1.5451	28	1.544	23	1.546
2	731	1.568	151	1.600	255	1.5771	743	1.578	368	1.578
3	931	1.607	45	1.631	189	1.6092	503	1.612	190	1.610
4	394	1.646	13	1.662	54	1.6413	246	1.647	93	1.642
5	222	1.685	12	1.693	37	1.6734	160	1.681	42	1.675
6	137	1.724	7	1.724	12	1.7055	106	1.716	29	1.707
7	80	1.763	3	1.756	12	1.7375	73	1.750	13	1.739
8	26	1.802	0	1.787	4	1.7696	30	1.785	4	1.771
9	14	1.841	0	1.818	2	1.8017	20	1.819	1	1.803
10	5	1.880	2	1.849	3	1.8338	6	1.854	2	1.835

The detail members (miRNAs) of the clusters using FDs for human, gorilla, chimpanzee, mouse and rat are given in the Supplementary Table S3. It is observed that the FD of miRNAs of human lies in the interval [1.50, 1.9] and the largest cluster (center at 1.60) contains 931 miRNAs, whereas the FD of miRNAs of gorilla lies in the interval [1.55, 1.86] with the largest cluster (center at 1.60) of miRNAs of gorilla that contains 151 miRNAs and the FD of miRNAs of chimpanzee lies in the interval [1.52, 1.84] with the largest cluster (center at 1.58) that contains 255 miRNAs. For the species mouse and rat, it is found that the FD of miRNAs of mouse lies in the interval [1.52, 1.87] and the largest cluster (center at 1.57) contains 743 miRNAs. The FD of miRNAs of rat lies in the interval [1.53, 1.85] and the largest cluster (center at 1.57) contains 368 miRNAs. It is worth mentioning that all the four intervals of FDs of the four species (except human) are contained in the interval of FD [1.50, 1.9] of human.

The centers of largest FD clusters of miRNAs of human, gorilla are approximately same (1.60) that reflect they are evolutionarily close. Further, the centers of largest clusters of miRNAs of chimpanzee, mouse and rat are approximately same (1.57) which is a reflection of the fact that chimpanzee, mouse and rat species are evolutionarily close. It is noted that there is no miRNAs of gorilla whose FD lies in between 1.76 and 1.84 whereas there are approximately 72 miRNAs of human and 7 miRNAs of chimpanzee whose FD lies in the said interval. There are clusters (for human, gorilla and chimpanzee, mouse and rat) with largest centers among the other centers of the clusters contain 5, 2, 3, 6 and 2 members respectively.

### Classification Based on HEs

**Figure 4:**
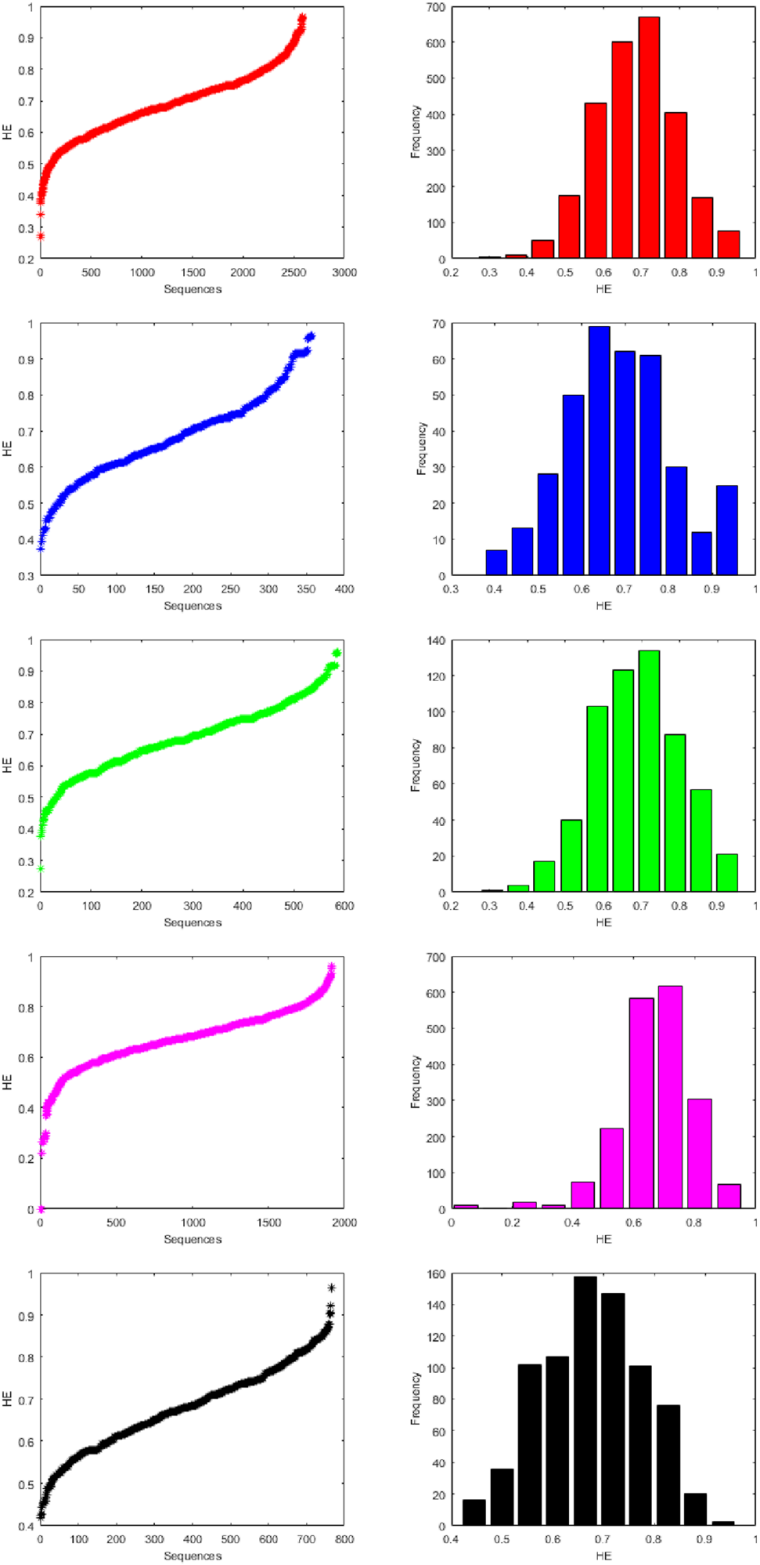
Histograms of Hurst exponents of miRNAs of Human, Gorilla, Chimpanzee, Mouse and Rat from top to bottom respectively.

**Figure 5:**
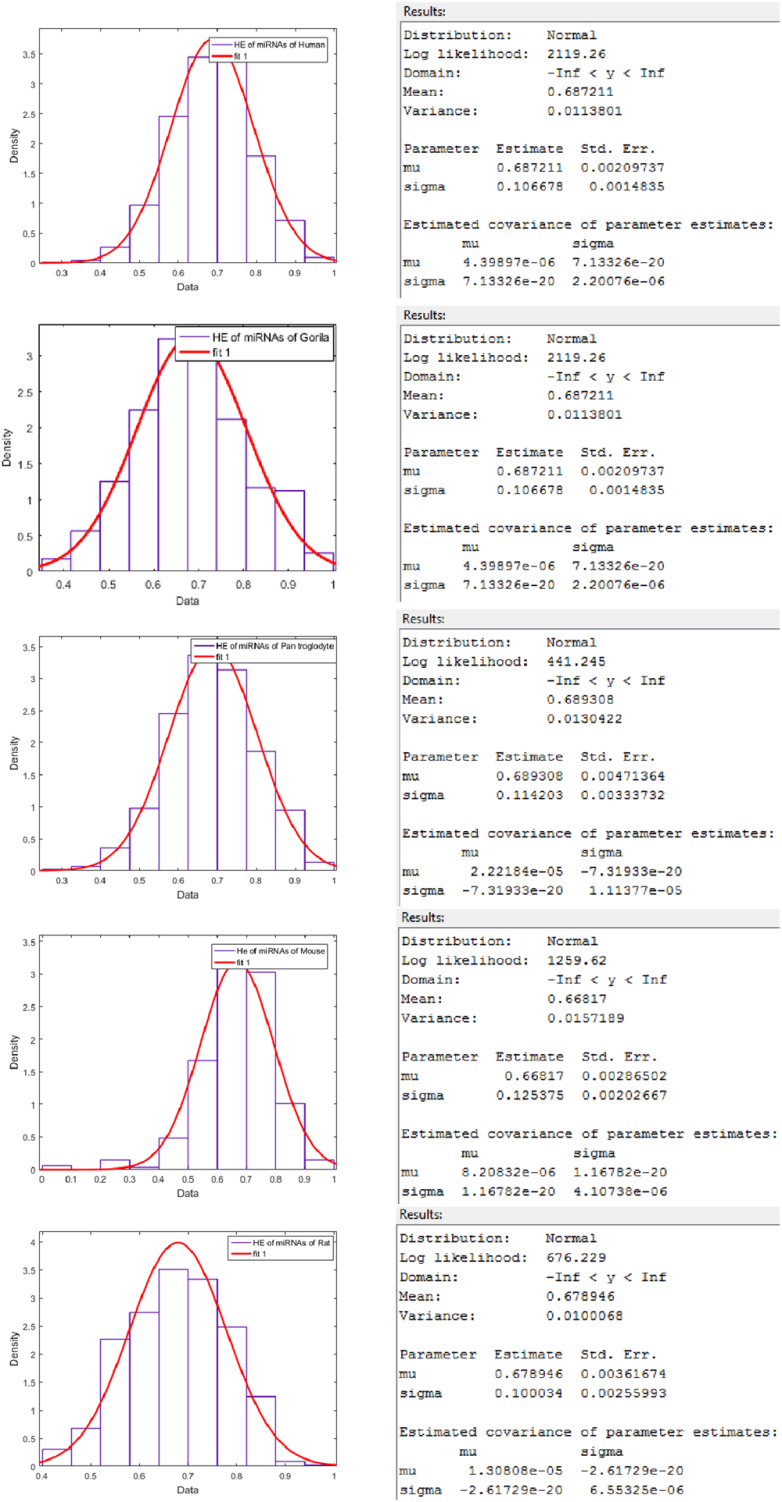
Normal distribution fitting over HEs of miRNAs of Human, Gorilla, Chimpanzee, Mouse and Rat from top to bottom respectively.

For each binary sequence of miRNA of human, gorilla, chimpanzee, mouse and rat, the Hurst exponent (using Equation (3)) is determined and then a classification is made which is shown in Table 3 for all the species. The Hurst exponents and the histograms of all the miRNAs five species are plotted in the Fig. 4. Also a normal distribution fitting is also made as shown in Fig. 5.

**Table 3:** Clusters based on Hurst exponent of miRNAs of Human, Gorilla, Chimpanzee, Mouse and Rat.

Cluster	Human		Gorilla		Chimpanzee		Mouse		Rat	
	Frequency	Center	Frequency	Center	Frequency	Center	Frequency	Center	Frequency	Center
1	3	0.301	7	0.404	1	0.309	11	0.048	16	0.445
2	11	0.371	13	0.463	4	0.377	0	0.144	36	0.500
3	50	0.441	28	0.522	17	0.446	20	0.240	102	0.554
4	173	0.510	50	0.581	40	0.514	11	0.336	107	0.609
5	430	0.580	69	0.640	103	0.583	74	0.432	158	0.664
6	600	0.650	62	0.699	123	0.651	224	0.528	147	0.718
7	671	0.720	61	0.758	134	0.719	584	0.624	101	0.773
8	405	0.790	30	0.817	87	0.788	618	0.720	76	0.827
9	168	0.859	12	0.876	57	0.856	305	0.816	20	0.882
10	76	0.929	25	0.935	21	0.925	68	0.912	2	0.936

The detail members (miRNAs) of the clusters for human, gorilla, chimpanzee, mouse and rat are given in the Supplementary Table S4. The HE of miRNAs of human lies in the interval [0.26, 0.96] and the largest cluster (center at 0.72) contains 671 miRNAs whereas the HE of miRNAs of gorilla lies in the interval [0.37, 0.96] and HE of miRNAs of chimpanzee in the interval [0.27, 0.96]. The largest cluster (center at 0.64) of miRNAs of gorilla contains 69 members and the same (center at 0.72) contains 134 miRNAs of chimpanzee. The HE of miRNAs of mouse lies in the interval [0.0, 0.96] and the largest cluster (center at 0.72) contains 618 miRNAs. The HE of miRNAs of rat lies in the interval [0.41, 0.96] and the largest cluster (center at 0.66) contains 158 miRNAs.

The centers of the largest HE clusters in the case of human, chimpanzee and mouse are close enough where as the center of the largest cluster of miRNAs of gorilla and rat is significantly different from other three species unlike FD as stated in the above section. It interprets basically the long range autocorrelations of miRNAs of gorilla and rat are significantly different from the miRNAs of human, chimpanzee and mouse. Therefore with regards to the centers of the largest HE clusters, we observed two sets of evolutionarily close species: human, chimpanzee and mouse (*HE* ≈ 0.72) belong to one set and, gorilla and rat belong to another set (*HE* ≈ 0.65).

### Classification Based on HDs

**Figure 6:**
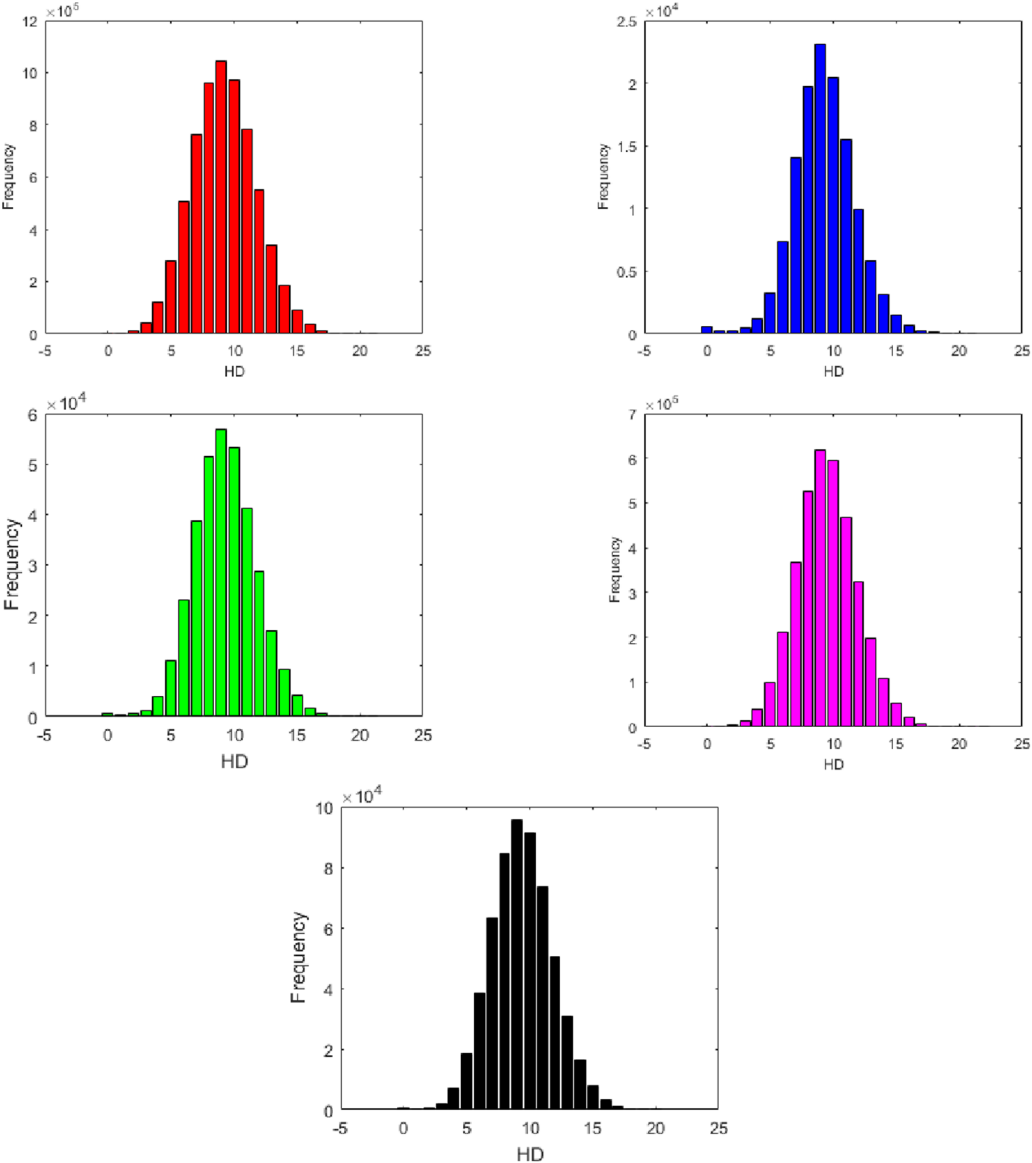
Bar plots of miRNAs of Human, Gorilla, Chimpanzee, Mouse and Rat based on hamming distances from top to bottom respectively.

The detail pairs of miRNAs based on minimum Hamming distances (using Equation (4)) of human, gorilla, chimpanzee, mouse and rat are given in the Supplementary Tables S5, S6, S7, S8 and S9 respectively. We then form classes of pairs of the binary strings (miRNAs) of the five species based on Hamming distances 0 to 22 with the percentages of each class as shown in Table 4. The bar plots of these class-frequencies are also given the Fig. 6.

**Table 4:** Clusters based on minimum Hamming distance of miRNAs of Human, Gorilla, Chimpanzee, Mouse and Rat.

H. Distance	Human		Gorilla		Chimpanzee		Mouse		Rat	
	N. of Pairs	%	N. of Pairs	%	N. Pairs	%	N. Pairs	%	N. of Pairs	%
0	3768	<1	557	<1	763	<1	2719	<1	805	<1
1	2602	<1	206	<1	330	<1	2034	<1	158	<1
2	10620	<1	244	<1	572	<1	4934	<1	462	<1
3	39956	1	436	<1	1264	<1	13496	<1	1872	<1
4	119160	2	1154	1	4052	1	39704	1	6990	1
5	276592	4	3274	3	10996	3	100152	3	18498	3
6	505864	8	7370	6	23074	7	211352	6	38632	7
7	760868	11	14014	11	38752	11	367670	10	63188	11
8	960910	14	19714	15	51488	15	525746	14	84410	14
9	1042752	16	23076	18	56794	16	617676	17	95526	16
10	971648	15	20408	16	53210	15	594428	16	91106	16
11	781550	12	15476	12	41256	12	469378	13	73542	13
12	549660	8	9930	8	28722	8	324030	9	50378	9
13	339810	5	5844	5	17082	5	198398	5	30910	5
14	186330	3	3144	2	9332	3	108954	3	16364	3
15	89458	1	1444	1	4244	1	52832	1	7702	1
16	37768	1	720	1	1728	1	21986	1	3178	1
17	13404	<1	284	<1	646	<1	7930	<1	1068	<1
18	3918	<1	144	<1	200	<1	2388	<1	328	<1
19	900	<1	6	<1	54	<1	784	<1	90	<1
20	174	<1	2	<1	8	<1	402	<1	18	<1
21	32	<1	2	<1	2	<1	184	<1	—	—
22	—	—	—	—	—	—	48	<1	—	—

Here number of pairs for each hamming distance and the corresponding percentage (approx.) are shown.

For all the species except rat, none of the clusters with Hamming distances from 0 to 21 is empty. In the case of mouse only, there are 48 pairs of miRNAs having *HD* = 22. It is also seen that the largest clusters with HD 9 for miRNAs of human, gorilla, chimpanzee, mouse and rat contain 1042752, 23076, 56794, 617676 and 95526 number of pairs respectively. It interprets that the arrangement of the purine and pyrimidine bases for most of miRNAs of human, gorilla, chimpanzee, mouse and rat are differed by 9 bases only.

### Classification Based on Distance Pattern of Purine and Pyrimidine

**Figure 7:**
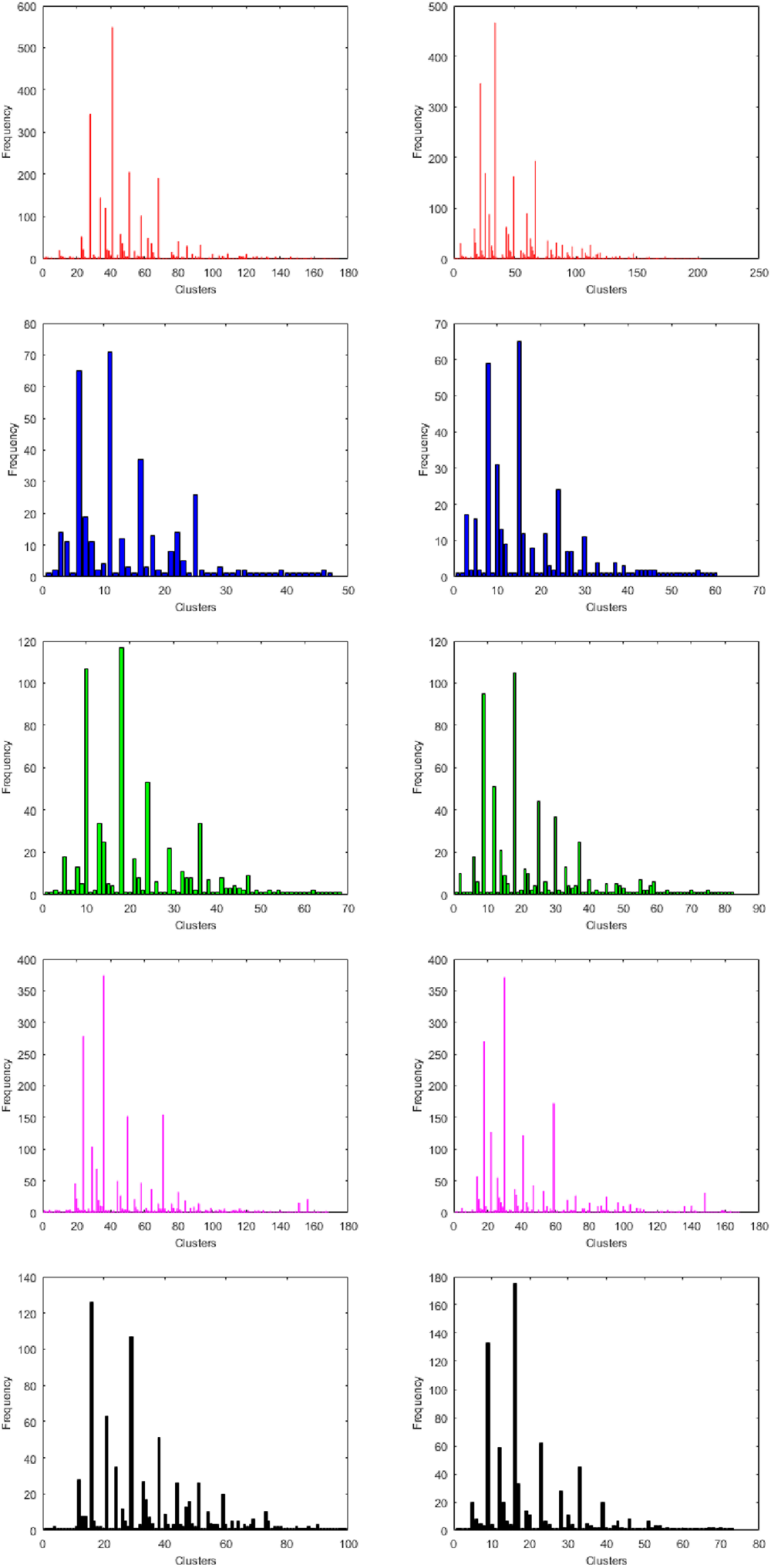
Bar plots of purine (on top) and pyrimidine (on bottom) distances of miRNAs of Human, Gorilla, Chimpanzee, Mouse and Rat from left to right respectively.

For all the miRNAs of five species, the distance patterns between purine bases to the next immediate purine bases are obtained. There are 174, 47, 68, 168 and 99 clusters based on unique distinct patterns of purine bases distance (gap) of miRNAs of human, gorilla, chimpanzee, mouse and rat are shown in Supplementary Table S10. For an example, the pattern of purine distances in the miRNAs of *h*421 of human is [1-2-3-4-12] of which is interpreted as there are purine bases which are 1, 2, 3, 4 and 12 bases apart. The bar plot of different clusters frequencies (number of miRNAs) for the five species human, gorilla, chimpanzee, mouse and rat are plotted in Fig. 7.

It is found that most of the miRNAs have purine distance patterns [1-2-3] and [1-2-3-4]. There are exactly 549 and 343 many miRNAs of human, 71 and 65 miRNAs of gorilla, 117 and 107 miRNAs of chimpanzee, 374 and 278 miRNAs of mouse, 107 and 126 of miRNAs of rat for the purine distance pattern [1-2-3] and [1-2-3-4] respectively. There is no miRNAs of gorilla, chimpanzee and rat having purine distance pattern [1]. In all the five cases, it is noted that there are several clusters having only one member which means that the miRNA of those clusters have unique purine distance pattern. In the similar fashion, for all the miRNAs of human, gorilla, chimpanzee, mouse and rat, the distance between pyrimidine bases to the next immediate pyrimidine bases are found, which is tabulated in the Supplementary Table S10. The maximum length of the purine and pyrimidine distance patterns is found to be 5 for all the five sets of miRNAs except only five miRNAs of human.

**Figure 8:**
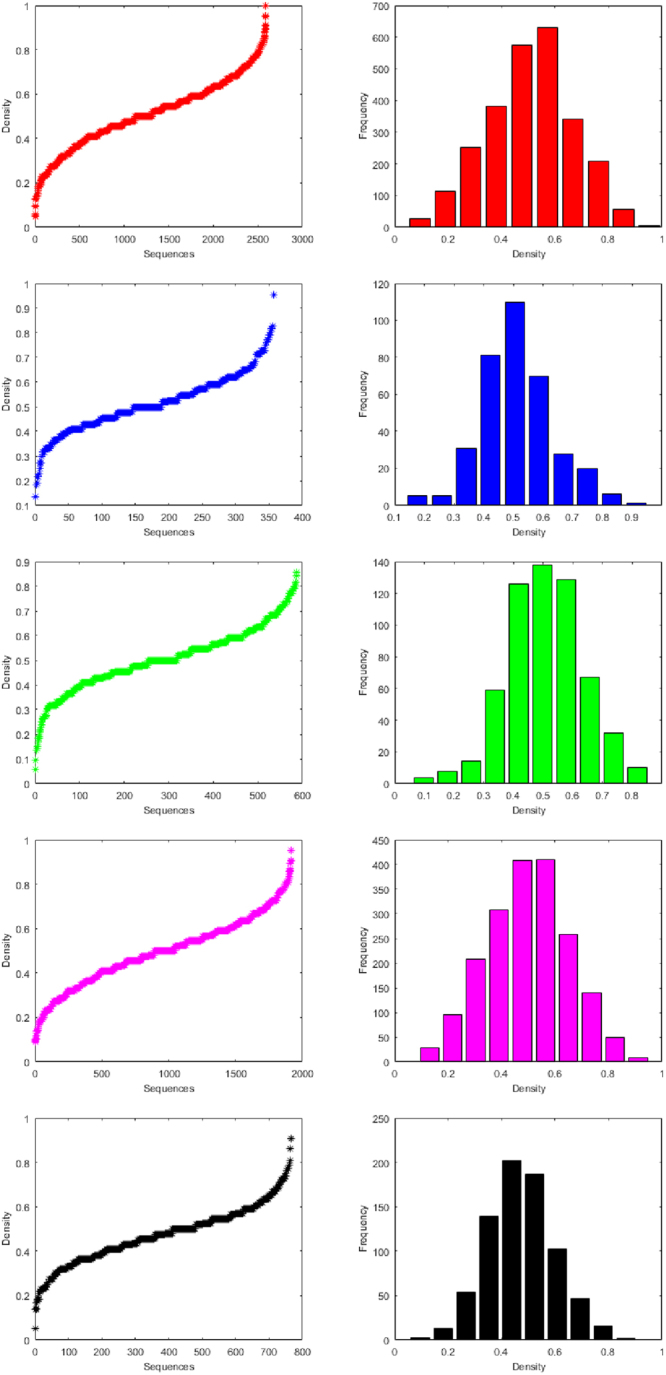
Histograms of frequency of Density for purine bases of the miRNAs of Human, Gorilla, Chimpanzee, Mouse and Rat from top to bottom respectively.

We also have determined the density of purine and pyrimidine bases of the miRNAs of human, gorilla, chimpanzee, mouse and rat as presented in detail in the Supplementary Table S11. If the length of the miRNA is 20 (20 nt) in which number of purine bases (1’s) is 8 and number of pyrimidine bases (0’s) is 12, then the density of purine is 0.4 and density of pyrimidine is 0.6. The clusters based on the density of purine is made and tabulated in Table 5 for all the species. The histogram of the frequencies density for purine bases of all species are given in Fig. 8.

**Table 5:** Clusters based on Density (Purine) of miRNAs of Human, Gorilla, Chimpanzee, Mouse and Rat.

Cluster	Human		Gorilla		Chimpanzee		Mouse		Rat	
	Frequency	Center	Frequency	Center	Frequency	Center	Frequency	Center	Frequency	Center
1	27	0.095	5	0.177	4	0.099	29	0.130	3	0.095
2	114	0.190	5	0.259	8	0.179	96	0.217	13	0.181
3	253	0.286	31	0.341	14	0.258	209	0.304	54	0.267
4	381	0.381	81	0.423	59	0.338	308	0.391	139	0.352
5	575	0.476	110	0.505	126	0.418	408	0.477	202	0.438
6	628	0.571	70	0.586	138	0.498	410	0.564	187	0.524
7	340	0.667	28	0.668	129	0.578	258	0.651	102	0.609
8	208	0.762	20	0.750	67	0.658	139	0.738	47	0.695
9	57	0.857	6	0.832	32	0.737	50	0.824	16	0.781
10	5	0.952	1	0.914	10	0.817	8	0.911	2	0.866

### Classification Based on SEs

**Figure 9:**
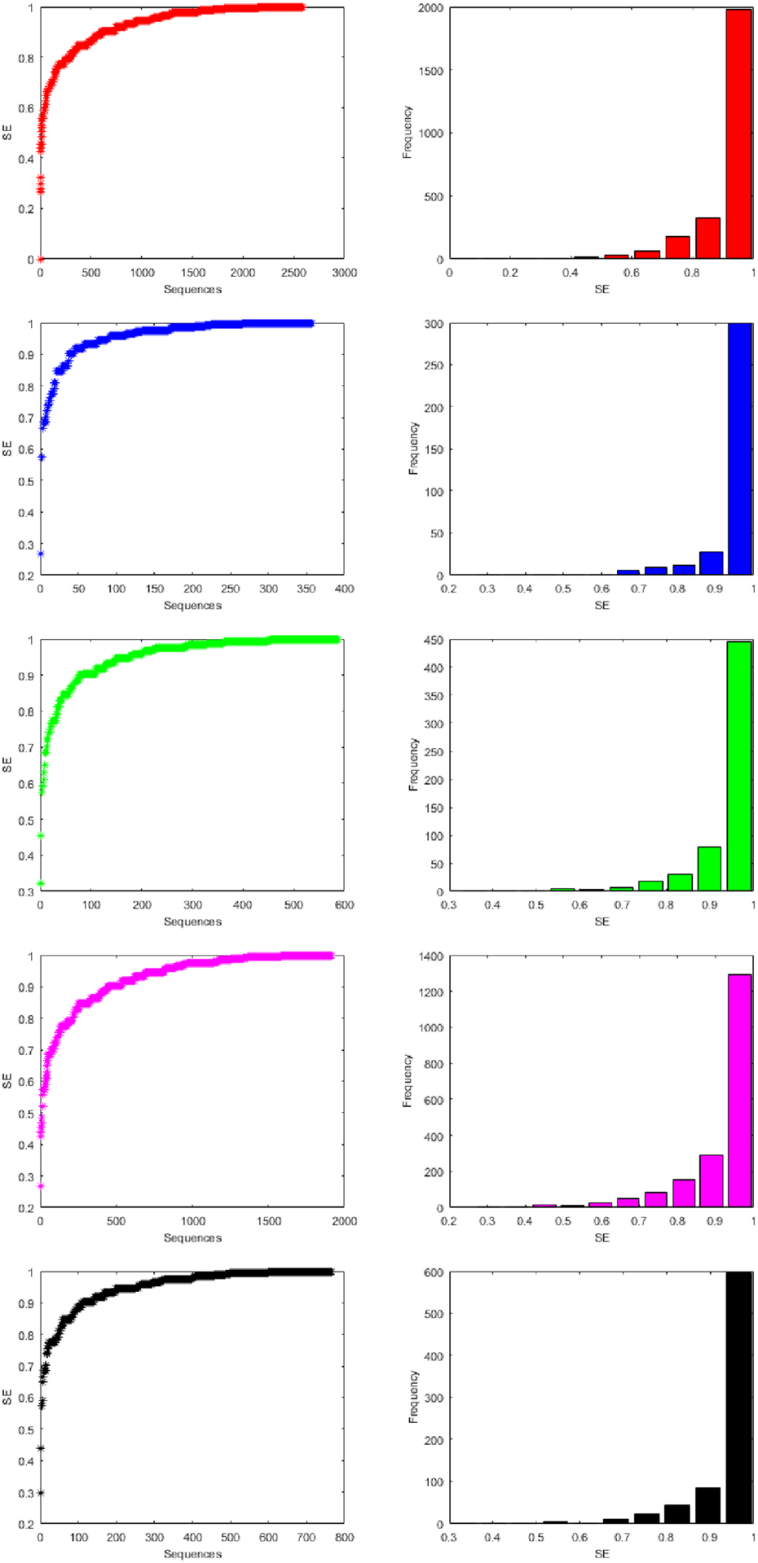
Histograms of Shannon entropy (purine and pyrimidine) Human, Gorilla, Chimpanzee, Mouse and Rat from top to bottom respectively.

For all the miRNAs of the five species, the Shannon entropy (using Equation (5)) is calculated of which detail can be seen in the Supplementary Table S12. In the case of miRNAs of human, there are exactly 80 distinct SE values are obtained whereas in the case of miRNAs of gorilla, chimpanzee, mouse and rat there are 38, 57, 73 and 55 respectively distinct SEs are found. Based on the Shannon entropy, the miRNAs of the five species are classified into 10 clusters separately as shown in the Table 6. The histograms of SEs of all the miRNAs of human, gorilla, chimpanzee, mouse and rat are plotted in the Fig. 9.

**Table 6:** Clusters based on Shannon entropy of miRNAs of Human, Gorilla, Chimpanzee, Mouse and Rat.

Cluster	Human		Gorilla		Chimpanzee		Mouse		Rat	
	Frequency	Center	Frequency	Center	Frequency	Center	Frequency	Center	Frequency	Center
1	1	0.303	1	0.303	1	0.357	1	0.303	1	0.333
2	0	0.377	0	0.377	1	0.424	0	0.377	0	0.403
3	4	0.450	0	0.450	0	0.492	12	0.450	1	0.473
4	1	0.523	0	0.523	4	0.560	6	0.523	3	0.543
5	9	0.597	1	0.597	3	0.628	24	0.597	1	0.614
6	29	0.670	6	0.670	7	0.695	51	0.670	10	0.684
7	65	0.743	9	0.743	17	0.763	82	0.743	24	0.754
8	179	0.817	12	0.817	30	0.831	152	0.817	44	0.824
9	323	0.890	28	0.890	78	0.898	291	0.890	84	0.895
10	1977	0.963	300	0.963	446	0.966	1296	0.963	597	0.965

It is observed that most of the miRNAs of all the species human, gorilla, chimpanzee, mouse and rat are having Shannon entropy centered at 0.96 which is the largest center of the clusters for all the three different clustering, which contain 1977, 300, 446, 1296 and 597 miRNAs respectively. It is seen that there is no member having SE 0.5 of all the miRNAs in all the five species human, gorilla, chimpanzee, mouse and rat. It interprets that none of the miRNAs is having equal (approximately) purine-pyrimidine density over the sequences. Overall these observations draw an impression that almost none of the miRNAs of all the five species are having random-like purine-pyrimidine distributions.

## Discussion

The purine and pyrimidine analysis with the binomial distribution shows the purines and pyrimidines are not independently distributed over the miRNAs and there is a tendency of same properties (purine or pyrimidine) to repeat in a miRNA. We find the various classes using different methods where most of the cases the classes are normally distributed although the distribution of the purines and pyrimidines is not random like distribution.

There are 5 miRNAs of human in the cluster 10 based on fractal dimension as shown in the Table 2 having maximum FD. We have seen closely those sequences and find that three of them (h2248, h1954 and h2552) are pyrimidine rich sequences (94%, 90% and 90% respectively) and the other two (h1835 and h1291) are purine rich sequences (95% and 100%) as shown in Table 7. In the case of miRNAs of gorilla, chimpanzee, mouse and rat the cluster 10 contains 2, 3, 6 and 2 miRNAs respectively. All these miRNAs of gorila, chimpanzee, mouse and rat are either purine or pyrimidine rich as shown in Table 7. Based on the observations here we strongly suggest that whenever the amount of purine or pyrimidine is quite high in a miRNA sequence, then the corresponding FD will be maximum.

**Table 7:** MiRNAs (from the cluster-10 based on fractal dimension) and its density distribution of miRNAs of the five species.

Encripted Name	miRNAs sequence	Purine Density	Pyrimidine Density
h2248	UGCCCUCCUUUCUUCCCUC	0.053	0.947
h1954	CUUCUCUUCUCUCCUUCCCAG	0.095	0.905
h2552	UCCUCUUCUCCCUCCUCCCAG	0.955	0.045
h1835	AAGACGGGAGGAAAGAAGGGAG	0.095	0.905
h1291	GGGAAAAGGAAGGGGGAGGA	1	0
g67	ACGCCCUUCCCCCCCUUCUUCA	0.14	0.86
g12	AAGACGGGAGGAAAGAAGGGAG	0.95	0.045
p527	UCGCCUCCUCCUCUCCC	0.05	0.95
p460	CCUCUUCCCCUUGUCUCUCCA	0.095	0.905
p205	ACGCCCUUCCCCCCCUUCUUCA	0.14	0.86
m1110	ACGCCCUUCCCCCCCUUCUUCA	0.14	0.86
m1606	UCUCUCCUUUCUCCUCCUAG	0.1	0.9
m1871	UUUCUUCUCUUCCCUUUCAG	0.1	0.9
m1105	AAAAGGGAGAGAAAGAAAUGC	0.905	0.095
m1091	AAGACGGGAGAAGAGAAGGGAG	0.95	0.045
m768	UGUCCUCUUCUCCCUCCUCCCA	0.091	0.909
r711	GGUCUUGUUUGGGUUUGUU	0.052	0.947
r455	ACAACAACACCAAACCACCUGA	0.909	0.091

There are several clusters having miRNAs for human, gorilla, chimpanzee, mouse and rat with the same HEs. The density distribution of purine and pyrimidine are balanced for all such miRNAs having same HEs. For an example, we took 17 miRNAs from the cluster 7 of miRNAs of human based on Hurst exponent (h463, h526, …, h1824 and h2202) which are all having the same HE (0.714484) as shown in the Table 8. It is found that the density distributions of purine and pyrimidine are 60% and 40% (or 40% and 60%) respectively. It is also seen that these 17 miRNAs of human belong to same cluster 3 based on fractal dimension. It is observed that there are 0.8% miRNAs of human, 1.1% of gorilla, 0.68% of chimpanzee, 0.67% of mouse and 0.4% of rat miRNAs which are having the same HE (0.5) indicating a completely uncorrelated purine and pyrimidine spatial ordering over the miRNAs. This investigation reassures that the miRNAs for all the five species are deviating from randomness. It is also noted that there are exactly 11 miRNAs only of mouse having zero Hurst exponent that interprets those 11 miRNAs are having consecutive purines (pyrimidines) and pyrimidines (purines).

**Table 8:** MiRNAs (from the cluster-7 based on Hurst exponent) and its FD with the density distribution of miRNAs of Human.

miRNAs of human	HE	FD	Purine Density	Pyrimidine Density
h463	0.714483912	1.611	0.409	0.591
h526	0.714483912	1.608	0.409	0.591
h1655	0.714483912	1.615	0.591	0.409
h1733	0.714483912	1.608	0.591	0.409
h2161	0.714483912	1.612	0.591	0.409
h2219	0.714483912	1.604	0.591	0.409
h262	0.714483912	1.599	0.591	0.409
h515	0.714483912	1.588	0.591	0.409
h747	0.714483912	1.592	0.409	0.591
h782	0.714483912	1.590	0.591	0.409
h1040	0.714483912	1.583	0.409	0.591
h1061	0.714483912	1.583	0.591	0.409
h1240	0.714483912	1.604	0.591	0.409
h1348	0.714483912	1.590	0.591	0.409
h1680	0.714483912	1.586	0.409	0.591
h1824	0.714483912	1.582	0.591	0.409
h2202	0.714483912	1.586	0.409	0.591

Now we see the miRNAs of human having identical distance patterns of purine and pyrimidine. It is observed that there are very few numbers of miRNAs of human which are having identical distance pattern of purine and pyrimidine. For example, h61 miRNA is having identical distance pattern of purine and pyrimidine [1-2]. Also There are miRNAs h36, h51, h62, h83, h122 and h2584 having identical distance pattern of purine and pyrimidine [1-2-3]. There are miRNAs of gorilla g6, g13, g19, g20 and g21 having identical distance pattern of purine and pyrimidine [1-2-3-4]. In the set of miRNAs of chimpanzee, there are p55, p84, p106, p119 and p138 having identical purine and pyrimidine distance pattern [1-2-3-4]. Similar distance patterns are also seen in the miRNAs of mouse and rat. These identical distance patterns of purine and pyrimidine make a guarantee that there are purine and pyrimidine blocks of same length.

There are 142, 58, 92, 126 and 88 distinct densities of purine and pyrimidine bases across miRNAs of human, gorilla, chimpanzee, mouse and rat are found. Out of 2588 miRNAs of human, 194 miRNAs of human having equal density (0.5), 1121 miRNAs of human having lesser density (less than 0.5) of purines than that of pyrimidine, 1273 miRNAs of human having higher density (greater than 0.5) of purine than that of pyrimidine. This infers density of pyrimidine is richer than that of purine over the set of miRNAs of human. It is found that there are 43 miRNAs out of 357 miRNAs of gorilla, 67 out of 587 miRNAs of chimpanzee, 154 out of 1915 miRNAs of mouse and 69 out of 765 miRNAs of rat having equal density (0.5) of purine and pyrimidine. There are 146 miRNAs over 357 miRNAs of gorilla, 268 miRNAs over 587 miRNAs of chimpanzee, 893 miRNAs over 1915 miRNAs of mouse and 411 miRNAs over 765 of rat having lesser density of purines than that of pyrimidine. In this regard we infer that the densities of pyrimidine over the miRNAs of these five observed species is richer than that of the purine bases. Out of all the 2588 miRNAs, the miRNA *hsa-miR-6124 MIMAT0024597* (h1291) is only miRNA containing all purine bases.

The evolutionary closeness among the species utilizing five parameters are shown in Table 9. For an example, the mean of FDs of 2588 miRNAs of human is 1.62 which is approximately same as for 1915 miRNAs of mouse. So, these two close species ({Human, Mouse}) are put in one set. Further, the mean of FDs of miRNAs of the species gorilla, chimpanzee and rat are close (≈1.60), so they are put in another set. Similarly, we have shown the close species based on the largest cluster center of each parameter in Table 9 as discussed in Result section.

**Table 9:** The set of evolutionarily close species based on mean quantitative value and largest cluster center of the discussed parameters. Pu-Purine.

Parameters	Close species w.r.t mean quantitative value	Close species w.r.t largest cluster Center
FD	{Human, Mouse} and {Gorilla, Chimpanzee, Rat}	{Human, Gorilla} and {Chimpanzee, Mouse, Rat}
HE	{Human, Gorilla, Chimpanzee}	{Human, Chimpanzee, Mouse} and {Gorilla, Rat}
HD	{Gorilla, Chimpanzee}	{Human, Chimpanzee, Rat}
Density of Pu	{Human, Chimpanzee}	{Human, Mouse} and {Gorilla, Chimpanzee}
SE	{Human, Mouse} and {Gorilla, Chimpanzee, Rat}	{Human, Gorilla, Chimpanzee, Mouse, Rat}

It is reported that MiR-200 (star miRNAs) is a family of tumour suppressor miRNAs consisting of five members (miR-200a-3p/h609, miR-200b-3p/h888, miR-200c-3p/h1520, miR-200a-429/h1670, miR-141-3p/h515), which are significantly involved in inhibition of epithelial-to-mesenchymal transition (EMT), repression of cancer stem cells (CSCs) self-renewal and differentiation, modulation of cell division and apoptosis, and reversal of chemoresistance^21^ as shown in Table 10 along with other four miRNAs of miR-200a-5p/h1449, miR-200b-5p/h1677, miR-200c-5p/h2186 and miR-141-5p/h328. We have chosen all these nine miRNAs of human including other miRNAs of human which are 0, 1 and 2 Hamming distance apart from those nine miRNAs.

**Table 10:** Star miRNAs (MiR-200) in human cancer and their quantifications. Pu-purine, Py-Pyrimidine.

HD	miRNAs	FD	HE	Pu Density	Py Density
	**h609**	**1.579**	**0.578**	**0.5**	0.5
HD = 1	h888	1.580	0.649	0.545	0.455
HD = 2	h515	1.588	0.714	0.591	0.409
HD = 2	h875	1.587	0.610	0.478	0.522
HD = 2	h1520	1.604	0.627	0.609	0.391
HD = 2	h1670	1.579	0.5	0.5	0.5
	**h888**	**1.580**	**0.649**	**0.545**	**0.455**
HD = 1	h515	1.588	0.714	0.5	0.5
HD = 1	h609	1.579	0.578	0.5	0.5
HD = 1	h1520	1.604	0.627	0.609	0.391
HD = 2	h1236	1.569	0.654	0.647	0.353
	**h1520**	**1.604**	**0.627**	**0.609**	**0.391**
HD = 1	h888	1.580	0.649	0.545	0.455
HD = 2	h515	1.588	0.714	0.591	0.409
HD = 2	h609	1.579	0.578	0.5	0.5
HD = 2	h2413	1.590	0.528	0.706	0.294
	**h1670**	**1.579**	**0.671**	**0.5**	**0.5**
HD = 2	h515	1.588	0.714	0.591	0.409
HD = 2	h609	1.579	0.578	0.5	0.5
HD = 2	h2485	1.533	0.873	0.471	0.529
	**h515**	**1.588**	**0.714**	**0.591**	**0.409**
HD = 1	h888	1.580	0.649	0.545	0.455
HD = 1	h1236	1.569	0.654	0.647	0.353
HD = 2	h609	1.579	0.578	0.5	0.5
HD = 2	h712	1.543	0.745	0.556	0.444
HD = 2	h782	1.590	0.714	0.591	0.409
HD = 2	h1520	1.604	0.627	0.609	0.391
HD = 2	h1670	1.579	0.671	0.5	0.5
	**h1449**	**1.579**	**0.671**	**0.5**	**0.5**
HD = 0	h1677	1.579	0.671	0.5	0.5
HD = 2	h749	1.509	0.655	0.438	0.563
HD = 2	h2186	1.596	0.638	0.409	0.591
	**h1677**	**1.579**	**0.671**	**0.545**	**0.455**
HD = 0	h1449	1.579	0.671	0.5	0.5
HD = 2	h749	1.509	0.655	0.438	0.563
HD = 2	h2186	1.601	0.638	0.409	0.591
	**h2186**	**1.596**	**0.638**	**0.409**	**0.591**
HD = 1	h749	1.509	0.655	0.438	0.563
HD = 2	h1449	1.579	0.671	0.5	0.5
HD = 2	h1677	1.579	0.671	0.545	0.455
	**h328**	**1.585**	**0.709**	**0.455**	**0.545**
HD = 0	_×_				
HD = 1	_×_				
HD = 2	_×_				

The FDs of five star miRNAs are almost similar except *h*1520 whose FD is slightly greater than the other four miRNAs. But the HEs are varying for all the five miRNAs. It is found that h888 is 1 Hamming distance apart from h609 and h1520 although h609 and h1520 are 2 Hamming distance apart. The miRNA h888 is having approximately same HD, HE and density of purine and pyrimidine bases with the miRNAs h609 and h1520. Hence we convict that h888 might also work as h609 and h1520 do. It is also observed that h1670 is 2 HD apart from the miRNAs h609 and h515 and the miRNA h1670 is showing very closeness as per quantitative measures and hence this miRNA h1670 would function as h609 and h515 do. As closeness is a transitive property (HD follows transitive inequality, *HD*(*a*, *b*) + *HD*(*b*, *c*) ≥ *HD*(*a*, *c*)), we can conclude that h888 could also function as h1670 (HD(h888, h1670) = 3). There are two miRNAs of human h1449 and h1677 which are 0 Hamming distance apart with same quantitative measures and hence we firmly propose that these two miRNAs would also function similarly. It is worth noting that both the miRNAs h1449 and h1677 have identical purine-pyrimidine organization. Following the similar argument, other association with the rest of miRNAs can also be made. It is seen that there does not exist any human miRNA which is 0, 1 or 2 HD apart from the miRNA h328. The five star miRNAs and their various combinations are associated with variety of diseases (*Table 1 in^21^ and^23,49–51^). The *miR*−200*a*−3*p*/*h*609 and *miR*−200*c*−3*p*/*h*1520 are associated with cancer type Cutaneous melanoma as reported in^52^. These two miRNAs are similar except in two bases (*HD* = 2) in their purine pyrimidine distribution.

In order to understand the association among the mRNAs and miRNAs, we take eight target mRNAs for some diseases and the set of associated miRNAs of human species as listed in Table 11 and Table 12. It is found that the FDs of mRNAs are quite higher than the same of associated miRNAs. The FDs of h150, h1617 and h22 are very close and they target to TGFBR2. It is observed that the density distribution of purine/pyrimidine is balanced for hh2220 and h616 and they target to DNMT3A causing the disease Lung Neoplasms. If we look for the distance pattern of purine and pyrimidine, we could observe some sub patterns for miRNAs to the corresponding mRNAs. The HD (using Equation (4)) between the purine pyrimidine distribution of miRNAs and the corresponding target mRNAs are determined and it turns out to be ranging from 1 to 5 (1 ≤ *HD* ≤ 5). This observation suggests that for some specific regions of target mRNAs of length around 22 nt, we have approximately (80–95)% similarities with the corresponding miRNAs.

**Table 11:** Selected miRNAs of Human and their corresponding target mRNAs. En-Encrypted name.

Sl no.	miRNAs	En	Target mRNAs	En	Disease
1	hsa-mir-143	h1697	ERK5	t1	Obesity
2	hsa-mir-20a	h150	TGFBR2	t2	Breast Neoplasms
3	hsa-mir-590	h1617	TGFBR2	t2	Carcinoma, Hepatocellular
4	hsa-mir-106a	h22	TGFBR2	t2	Colorectal Neoplasms
5	hsa-mir-16-1	h691	BCL2	t3	Leukemia, Lymphocytic, Chronic, B-Cell
6	hsa-mir-15a	h2533	BCL2	t3	Leukemia, Lymphocytic, Chronic, B-Cell
7	hsa-mir-221	h1013	KIT	t4	Thyroid Neoplasms
8	hsa-mir-146a	h1057	KIT	t4	Thyroid Neoplasms
9	hsa-mir-200a	h609	RAB30	t5	Carcinoma, Hepatocellular
10	hsa-mir-103a-2	h29	DMPK	t6	Myotonic Dystrophy
11	hsa-mir-29a	h2220	DNMT3A	t7	Lung Neoplasms
12	hsa-mir-29b-1	h616	DNMT3A	t7	Lung Neoplasms
13	hsa-mir-185	h2277	DNMT3A	t7	Glioma
14	hsa-mir-30c	h2299	KRAS	t8	Breast Cancer

**Table 12:** Target mRNAs/miRNAs and corresponding quantifications. Pu-Purine, Py-Pyrimidine, Dp-Distance pattern.

Target mRNAs or miRNAs	FD	HE	Pu Density	Py Density	Pu-Dp	Py-Dp
ERK5/t1	1.94	0.66	0.47	0.53	[1-2-3-4-5-6-7-8-9-10-11]	[1-2-3-4-5-6-7-8-9-10-12-13]
has-mir-143/h1697	1.590	0.745	0.571	0.429	[1-2-3]	[1-2-3-5]
TGFBR2/t2	1.8	0.57	0.53	0.47	[1-2-3-4-5-6-7-8-9-10]	[1-2-3-4-5-6-7-8-9-10-11-12-17]
has-mir-20a/h150	1.579	0.633	0.545	0.455	[1-2-3-4]	[1-2-4]
has-mir-590/h1617	1.570	0.569	0.476	0.524	[1-2-3-5]	[1-2-3-4]
has-mir-106a/h22	1.580	0.695	0.455	0.545	[1-2-5]	[1-2-5]
BCL2/t3	1.88	0.68	0.49	0.51	[1-2-3-4-5-6-7-9-13]	[1-2-3-4-5-6-7-8-10]
has-mir-16-1/h691	1.584	0.472	0.455	0.545	[1-2-3]	[1-2-3]
has-mir-15a/h2533	1.599	0.575	0.409	0.591	[1-2-3-5]	[1-2-4]
KIT/t4	1.92	0.58	0.52	0.48	[1-2-3-4-5-6-7-8-9-10-13]	[1-2-3-4-5-6-7-8-9-10-11-12-14-16]
has-mir-221/h1013	1.611	0.540	0.391	0.609	[1-2-3-4]	[1-2-4]
has-mir-146a/h1057	1.608	0.723	0.364	0.636	[1-4-7]	[1-3-5]
RAB30/t5	1.87	0.59	0.54	0.46	[1-2-3-4-5-6-7-8-10-13]	[1-2-3-4-5-6-7-8-9-14-15]
has-mir-200a/h609	1.579	0.578	0.500	0.500	[1-2-3-4]	[1-2-3]
DMPK/t6	1.9	0.64	0.52	0.48	[1-2-3-4-5-6-7-8-9-10-14]	[1-2-3-4-5-6-7-8-9-10-12]
has-mir-103a-2/h29	1.643	0.668	0.348	0.652	[1-2-3-5-8]	[1-2-3]
DNMT3A/t7	1.91	0.66	0.60	0.40	[1-2-3-4-5-6-7-9]	[1-2-3-4-5-6-7-8-9-10-11-13]
has-mir-29a/h2220	1.578	0.578	0.500	0.500	[1-2-3-4]	[1-2-3-5]
has-mir-29b-1/h616	1.597	0.557	0.500	0.500	[1-2-3-4-5]	[1-2-3]
has-mir-185/h2277	1.621	0.839	0.409	0.591	[1-3-10]	[1-3]
KRAS/t8	1.9	0.61	0.54	0.46	[1-2-3-4-5-6-7-8-9]	[1-2-3-4-5-6-7-8-9-10-11-12-13]
has-mir-30c/h2299	1.587	0.916	0.500	0.500	[1-3-4]	[1-2-10]

Through these investigations based on quantitative measures, we observe that their is no direct association among the miRNAs and target mRNAs due to their many-many relationships. The complex relationship among miRNAs and the target mRNAs is very much dynamic under various specific conditions as previously pointed out in the literatures^13,49,51^. Thus it has also been realized through our quantitative analysis over a set of miRNAs and target mRNAs. Our analyses can presume a set of possible miRNAs which would play some key role on the target mRNAs as they are very close with regards to quantitative measures.

## Concluding Remarks

One of the integral divisions of nucleotides based on their chemical properties is purine-pyrimidine. We attempted to understand the distribution of purine and pyrimidine bases over all the miRNAs in five species human, gorilla, chimpanzee, mouse and rat. Quantitatively, we deciphered the self-organization of the purine and pyrimidine bases for all the miRNAs through the fractal dimension of the indicator matrix. Also we took out the auto correlation of purine-pyrimidine bases through the parameter Hurst exponent. To get the nearness of the miRNAs based on their purine-pyrimidine distribution, HD is employed. The purine-pyrimidine distance patterns including the frequency distribution have been found for all the miRNAs. For all these parameters, we did cluster the miRNAs into several clusters. Based on the quantitative investigation, some crucial observations are adumbrated in the discussion. Our investigation over all the miRNAs of the five species through the purine and pyrimidine distributions triggers evolutionary closeness among the inter and intra families of different clusters.

Over the analysis through all the quantitative measures we could provide the set of miRNAs which relates target mRNAs and also the set of miRNAs that are associated with the specific diseases. Imperfect base-pairing between the miRNA and the 3′-UTR of its target mRNA leads to blockage of translation, or at least accumulation of the mRNA’s protein product, whereas perfect or near-perfect base-pairing between the miRNA and the middle of its target mRNA causes cleavage of the mRNA, thereby inactivating the same. Such diverse patterns of miRNAs may be responsible for making the correlation among miRNAs and target mRNAs complex, that are yet to be resolved decisively as pointed out by several researchers earlier. As, miRNAs are very smaller in size compared to their target mRNAs, the subsequence/subregions of specific mRNAs and miRNAs association might improve the results. In this context, we plan to bring out the patterns of organization of nucleotides following the other two modes of classifications (amino-keto and strong H-bond and weak H-bond) based on the chemical properties of nucleotides. This is in order to integrate the whole three kinds of grouping to find out the correlationship among the miRNAs-mRNAs and also the targeted regions of mRNAs.

## Electronic supplementary material

Dataset 1

Dataset 2

Dataset 3

Dataset 4

Dataset 5

Dataset 6

Dataset 7

Dataset 8

Dataset 9

Dataset 10

Dataset 11

Dataset 12
