# Supplementary material for: Analysis of Purines and Pyrimidines distribution over miRNAs of Human, Gorilla, Chimpanzee, Mouse and Rat
Source: Sci Rep. 2018 Jul 2;8:9974. doi: 10.1038/s41598-018-28289-x (PMC6028587; doi:10.1038/s41598-018-28289-x)
Supplement: Supplementary file 2 — Dataset 2 [file 41598_2018_28289_MOESM2_ESM.pdf]

1. Transformation of miRNAs (A/U/G/C) sequence to binary (0/1) sequence.

Input: Read the file (.tex) containing miRNAs sequences.

Example:
>hsa-miR-576-3p MIMAT0004796
AAGAUGUGGAAAAAUUGGAAUC
>hsa-miR-140-5p MIMAT0000431
CAGUGGUUUUACCCUAUGGUAG
>hsa-miR-522-5p MIMAT0005451
CUCUAGAGGGAAGCGCUUUCUG

Output: The file (named as humanmirans_transformed.txt') containing miRNAs sequences in binary format (A/G->1, U/C>0).

Example:
>hsa-miR-576-3p MIMAT0004796
1111010111111100111100
>hsa-miR-140-5p MIMAT0000431
0110110000100001011011
>hsa-miR-522-5p MIMAT0005451
0000111111111010000001

Process/Steps:
Step-1: Open and read the file.
Step-2: Get every sequence serially one by one.
Step-3: Read every nucleotide in the sequence and replaced by respective binary value.
Step-4: Write into the file.

```
% Codes in Matlab:
clc
clear;
fname='humanmirans';     % input file name
fid1=fopen(strcat(fname,'.txt'),'r');
fid2=fopen(strcat(fname,'_transformed.txt'),'w');  % output file name
tline1=fgets(fid1);
while ischar(tline1)
    fprintf(fid2,'%s',tline1);
    tline1=fgets(fid1);
    disp(tline1);
    td=strtrim(tline1);
    disp(length(td));
    str='';
    for i=1:length(td)
        if td(i)=='C' || td(i)=='G'
            str=strcat(str,'1');
        elseif td(i)=='U' || td(i)=='A' || td(i)=='T'
            str=strcat(str,'0');
        end
    end
    tline1=fgets(fid1);
    if ischar(tline1)
        fprintf(fid2,'%s\n',str);
    else
        fprintf(fid2,'%s',str);
    end
end
fclose(fid1);
fclose(fid2);
```

2. Finding the Fractal dimension (FD) of transformed miRNA sequence.

**Input:** Read the file (.tex) transformed miRNAs sequence file (binary format).
**Output:** An excel file containing FDs of miRNAs serially, histogram plot.
**Process/Steps:**<br>Step-1: Open and read the file.<br>Step-2: Get every sequence serially one by one.<br>Step-3: Obtained the indicator matrix using equation (1)).<br>Step-4: Calculate fractal dimension (FD) using (equation (2).<br>Step-5: Write FD into the file.

```
% Codes in Matlab:
clc
fname='humanmirnas';
fid1=fopen(strcat(fname,'_transformed.txt'),'r');
data=fgets(fid1);
FD=0;k=1;
while ischar(data)
    M1=0;
    data=strtrim(fgets(fid1)); disp(length(data));
    for i=1:length(data)
        for j=1:length(data)
            if data(i)==data(j)
                M1(i,j)=1;
            else
                M1(i,j)=0;
            end
        end
    end
    [len,len2]=size(M1);box=2;
    D=0;
    for box=2:len
    F=0;count=0;
    for i=1:len-(box-1)
    for j=1:len-(box-1)
            for k=i:i+(box-1)
                for l=j:j+(box-1)
                  if M1(k,l)==1
                      count=count+1;
                  end
                end
            end
    end
    end
    F=log10(count/((len-box+1)*(len-box+1)))/log10(box);
    D=D+F;
    end
    FD(k,1)=D/len;k=k+1;
    data=fgets(fid1);
end
fclose(fid1);
disp(FD);% here the variable FD contains the list of FDs for all sequences

sheet1 = 1;
xlRange = 'B2';
xlswrite(strcat(fname,'_fractal'),FD,sheet1,xlRange);

[i,j,k]=unique(FD,'rows');
OC2=histc(k,1:numel(j));
figure(1)
plot(sort(FD),'*','color','k');
xlabel('Sequences');
ylabel('FD');
hold on
figure(2)
[counts,centers] = hist(FD,10); % here 10 is the number of clusters
bar(centers,counts,'FaceColor','k');
xlabel('FD');
ylabel('Frequency');
```

3. **Finding the Hurst exponent (HE) of transformed miRNA sequence.**

**Input:** Read the file (.tex) transformed miRNAs sequence file (binary format).
**Output:** An excel file containing HEs of miRNAs serially, histogram plot.
**Process/Steps:**<br>Step-1: Open and read the file.<br>Step-2: Get every sequence serially one by one.<br>Step-3: Calculate Hurst exponent (HE) using equation (3).<br>Step-4: Write HE into the file.

```
% Codes in Matlab:
clc
fname='humanmirnas';
fid1=fopen(strcat(fname,'_transformed.txt'),'r');
data1=fgets(fid1);
HE=0;k=1;
while ischar(data1)
    data1=fgets(fid1);
    data1=strtrim(data1);
    HE(k)=hurst(data1);k=k+1;
    data1=fgets(fid1);
end
HE=transpose(HE);
disp(HE); % here the variable HE contains the list of HEs for all sequences

sheet1 = 1;
xlRange = 'B2';
xlswrite(strcat(fname,'_hurst_exponent'),HE,sheet1,xlRange);
figure(1)
plot(sort(HE),'*','color','k');
xlabel('Sequences');
ylabel('HE');
hold on
figure(2)
[counts,centers] = hist(HE,10); % here 10 is the number of clusters
bar(centers,counts,'FaceColor','k');
xlabel('HE');
ylabel('Frequency');
hold on
```

4. Finding the minimum hamming distance (HD) for each pair of transformed miRNA sequence.

**Input:** Read the file (.tex) transformed miRNAs sequence file (binary format).	
**Output:** An excel file containing HDs of miRNAs pair wise serially, histogram plot.	
**Process/Steps:**<br>Step-1: Open and read the file.<br>Step-2: Get two sequences serially pair wise one by one.<br>Step-3: Calculate the HD using equation (4).<br>Step-4: Write HD into the file.	

```
clc
fname1='humanmirnas';
fname2='humanmirnas';
fid1=fopen(strcat(fname1,'_transformed.txt'),'r');
data1=fgets(fid1);
k1=1;DM=0;count=0;
while ischar(data1)
    prevdata1=data1;
    temp1=strtrim(fgets(fid1));data1=temp1;
    len1=length(data1);
    fid2=fopen(strcat(fname2,'_transformed_.txt'),'r');data2=fgets(fid2);
    k2=0; disp(data2);
    while ischar(data2)
        k2=k2+1;
        prevdata2=data2; xxx=fgets(fid2);disp(xxx);
        temp2=strtrim(xxx);data2=temp2;
        len2=length(data2);
        if len1>=len2
            data1=temp1;data2=temp2;
        else
            data2=temp1;
            data1=temp2;
        end
        HD=min(len1,len2);
        for i=1:max(len1,len2)-min(len1,len2)+1
            d=0;
            for j=1:min(len1,len2)
                if (data1(i+j-1)=='1' && data2(j)=='1') || (data1(i+j-
                  1)=='0' && data2(j)=='0')
                else
                    d=d+1;
                end
            end
            if d<=HD
                HD=d;
            end
        end
        data2=fgets(fid2);
        DM(k1,k2)=HD; k1=k1+1;
    end
    fclose(fid2);
    data1=fgets(fid1);
end
fclose(fid1);
fclose(fid2);
disp(DM); % here the variable DM contains the list of HDs for all pair of
sequences

sheet1 = 1;
xlRange = 'B2';
xlswrite(strcat(fname,'_hamming_distances'),DM,sheet1,xlRange);

a=unique(DM);
out = [a,histc(DM(:),a)];
bar(out(:,1),out(:,2))
xlabel('HD');
ylabel('Frequency');
```

5. **Finding the Shannon entropy (SE) of transformed miRNA sequence.**

**Input:** Read the file (.tex) transformed miRNAs sequence file (binary format).
**Output:** An excel file containing SEs of miRNAs pair wise serially, histogram plot.
**Process/Steps:**<br>Step-1: Open and read the file.<br>Step-2: Get every sequence serially one by one.<br>Step-3: Calculate Shannon entropy (SE) using equation (5).<br>Step-4: Write SE into the file.

```
%Codes in Matlab:
clc
fname='humanmirnas';
fid1=fopen(strcat(fname,'_transformed.txt'),'r');
data1=fgets(fid1);
k=1;SE=0;
while ischar(data1)
    data1=strtrim(fgets(fid1));
    M=0; disp(length(data1));
    for i=1:length(data1)
        M(1,i)=str2num(data1(i));
    end
    [E,ia,ic] =unique(transpose(M),'rows','sorted');
    fr=histc(ic,1:numel(ia));
    [r,c]=size(ia);temp=0;
    for i=1:r
        p=fr(i)/length(data1);
        temp=temp+(p*log2(p));
    end
    SE(k,1)=-temp;k=k+1;
    data1=fgets(fid1);
end
fclose(fid1);
disp(SE); % here the variable SE contains the list of SEs for all sequences

sheet1 = 1;
xlRange = 'B2';
xlswrite(strcat(fname,'_Shanon_Entrophy'),SE,sheet1,xlRange);

figure(1)
plot(sort(SE),'*','color','k');
xlabel('Sequences');
ylabel('SE');
hold on
figure(2)
[counts,centers] = hist(SE,10); % here 10 is the number of clusters
bar(centers,counts,'FaceColor','k');
xlabel('SE');
ylabel('Frequency');
```

6. **Finding the Purine and pyrimidine distance pattern of transformed miRNA sequence.**

**Input:** Read the file (.tex) transformed miRNAs sequence file (binary format).
**Output:** An excel file containing pattern list of miRNAs serially, histogram plot.
**Process/Steps:**<br>Step-1: Open and read the file.<br>Step-2: Get every sequence serially one by one.<br>Step-3: Obtain the distance pattern (purine and pyrimidine).<br>Step-4: Write into the file.

%Codes in Matlab:

```
clc
fname='humanmirnas';
PT=cell(1,1);
fid1=fopen(strcat(fname,'_transformed.txt'),'r');
data=fgets(fid1);

k=1;P1=0;F1=0;P2=0;F2=0;Q1=0;Q2=0;
while ischar(data)
    d11=0;d22=0;d1=0;d2=0;f1=0;f2=0;temp1=0;temp2=0;i1=0;j1=0;k1=0;i2=0;
    j2=0;k2=0;
    data=fgets(fid1);data=strtrim(data);
    temp1=strfind(data,'1'); len1=length(temp1);
    temp2=strfind(data,'0'); len2=length(temp2);
    for i=1:len1-1
        d11(i)=temp1(i+1)-temp1(i);
    end
    for i=1:len2-1
        d22(i)=temp2(i+1)-temp2(i);
    end
    [p1,j1,k1]=unique(d11,'sorted');
    [p2,j2,k2]=unique(d22,'sorted');
    str='';
    for i=1:length(p1)
        if length(p1)==1
            str=strcat('[',num2str(p1(i)),']');
        elseif i==1
            str=strcat('[',str,num2str(p1(i)));
        elseif i==length(p1)
            str=strcat(str,'-',num2str(p1(i)),']');
        else
        str=strcat(str,'-',num2str(p1(i)));
        end
    end
    PT{k,1}=str;
    str='';
    for i=1:length(p2)
        if length(p2)==1
            str=strcat('[',num2str(p2(i)),']');
        elseif i==1
            str=strcat('[',str,num2str(p2(i)));
        elseif i==length(p2)
            str=strcat(str,'-',num2str(p2(i)),']');
        else
            str=strcat(str,'-',num2str(p2(i)));
        end
    end
    PT{k,2}=str;
    data=fgets(fid1);
    k=k+1;
end
fclose(fid);
```

```
disp(PT); % here the variable PT contains the list of purine and pyrimidine
distance pattern in two columns respectively for all sequences
sheet1 = 1;
xlRange = 'B2';
xlswrite(strcat(fname,'_distance_patterns'),PT,sheet1,xlRange);

Pu=PT(:,1);
Py=PT(:,2);
[C1,ia1,ic1] = unique(Pu);
[C2,ia2,ic2] = unique(Py);

out1 = [unique(ic1),histc(ic1,unique(ic1))];
bar(out1(:,1),out1(:,2),'FaceColor','b');
xlabel('Clusters');
ylabel('Frequency');

figure
out2 = [unique(ic2),histc(ic2,unique(ic2))];
bar(out2(:,1),out2(:,2),'FaceColor','b');
xlabel('Clusters');
ylabel('Frequency');
```
